# Biological and Social Predictors of HIV-1 RNA Viral Suppression in ART Treated PWLH in Sub-Saharan Africa

**DOI:** 10.3390/tropicalmed10010024

**Published:** 2025-01-16

**Authors:** Sindhuri Gandla, Raja Nakka, Ruhul Ali Khan, Eliezer Bose, Musie Ghebremichael

**Affiliations:** 1 Ragon Institute of MGH, MIT, and Harvard, Cambridge, MA 02139, USA; sgandla@mgh.harvard.edu (S.G.); rnakka@mgh.harvard.edu (R.N.); 2 Department of Mathematics, University of Arizona, Tucson, AZ 85721, USA; ruhul@math.arizona.edu; 3 Massachusetts General Hospital Institute of Health Professions, Boston, MA 02114, USA; elibose93@gmail.com; 4 Harvard Medical School, Massachusetts General Hospital, Cambridge, MA 02115, USA

**Keywords:** sub-Saharan Africa, HIV, machine learning, HIV-1 viral load

## Abstract

HIV remains a significant health issue, especially in sub-Saharan Africa. There are 39 million people living with HIV (PLWH) globally. Treatment with ART improves patient outcomes by suppressing the HIV RNA viral load. However, not all patients treated with ART suppress the HIV RNA viral load. This research paper explores the potential predictors of VL suppression in ART-treated PLWH. We used retrospective data from the 4820 ART-treated participants enrolled through population-based surveys conducted in Zambia and Malawi. We applied several machine learning (ML) classifiers and used the top classifiers to identify the predictors of VL suppression. The age of participants ranged from 15 to 64 years, with a majority being females. The predictive performance of the various ML classifiers ranged from 64% to 92%. In our data from both countries, the logistic classifier was among the top classifiers and was as follows: Malawi (AUC = 0.9255) and Zambia (AUC = 0.8095). Thus, logistic regression was used to identify the predictors of viral suppression. Our findings indicated that besides ART treatment status, older age, higher CD4 T-cell count, and longer duration of ART were identified as significant predictors of viral suppression. Though not statistically significant, ART initiation 12 months or more before the survey, urban residence, and wealth index were also associated with VL suppression. Our findings indicate that HIV prevention programs in the region should integrate education on early ART initiation and adherence in PLWH.

## 1. Introduction

Human Immunodeficiency Virus (HIV) has become a critical global health issue, impacting millions of people worldwide. It targets and weakens the body’s immune system by attacking CD4 T-cells, essential for immune defense. If not treated, it can lead to Acquired Immunodeficiency Syndrome (AIDS), a condition where the immune system is compromised, making the body vulnerable to opportunistic infections [1]. HIV disease progression can be monitored by measuring the HIV-1 Ribonucleic Acid (RNA) viral load (VL), calculated in copies per milliliter of blood [2]. Treatment with Antiretroviral Therapy (ART) can significantly reduce the amount of HIV in the blood, leading to viral suppression, defined as ≤1000 HIV-RNA copies/mL per World Health Organization (WHO) criteria [3]. Through ART, people living with HIV (PLWH) can also achieve a good life span [4]. The HIV virus relies on CD4 T-cells to replicate and transmit. Thus, both the CD4 T-cell count and the HIV RNA viral load are frequently used to monitor HIV disease progression and treatment efficacy [5].

UNAIDS has set a target of reaching the 95-95-95 goal by 2025, which means that by 2025, 95% of people with HIV will know their HIV status, 95% of people with HIV will receive ART, and 95% of people receiving ART will have VL suppression [6]. Globally, among the 39 million PLWH in 2022, only 29.8 million people were accessing ART, and 71% of those people receiving ART achieved viral suppression [7]. Globally, there has been a more than three-fold increase in the number of people receiving ART in the last 10+ years [7]. An increase in ART usage was also seen in sub-Saharan Africa, where HIV/AIDS continues to be a public health burden. In sub-Saharan Africa, the percentage of PLWH receiving ART has increased from 66% to 83% from 2017 to 2022. Of the 83% who received ART, 77% have achieved VL suppression [8]. A closer examination of country-specific data from Malawi and Zambia, the two countries our datasets are drawn from, reveals similar trends. According to the UNAIDS report, Malawi had 1.0 million PLWH, with 94% knowing their status, of them 93% on treatment, and of the treated, 87% achieved viral suppression [8]. Similarly, in Zambia, 1.4 million people were living with HIV in 2022, with 93% knowing their status and of those aware, 90% were on ART, and of those treated, 87% were virally suppressed [8]. Despite improvements, these figures highlight that we are still far from achieving the UNAIDS target of 95% viral suppression by 2025.

In sub-Saharan Africa, efforts to achieve the UNIADS goal have led to the implementation of several prevention strategies, including widespread testing, the use of ART, and educational programs aimed at reducing stigma and creating more awareness [9,10,11]. The educational programs have helped increase the number of people identifying their HIV status and receiving ART treatment [10,11]. Despite these efforts, numerous challenges remain in the region, which has limited access to treatment and healthcare services. To combat the HIV epidemic, Zambia and Malawi have adopted and implemented World Health Organization (WHO) guidelines aimed at reducing HIV-related morbidity. Both countries have embraced the WHO “Treat All” approach starting in 2016, which promotes the early initiation of ART through rapid HIV testing [12]. This approach focuses on providing ART to all individuals diagnosed with HIV, regardless of their CD4 count, clinical stage, or age, to curb new infections and deliver essential care [12]. According to these guidelines, viral load (VL) testing is conducted at six and twelve months after ART initiation and annually thereafter [13]. The WHO guidelines recommend a preferred combination of antiretroviral (ARV) drugs, including tenofovir, lamivudine, emtricitabine, and efavirenz, as the first-line regimen for ART treatment [13]. Patients with a VL exceeding 1000 copies/mL are enrolled in enhanced adherence counseling sessions, which aim to address issues affecting ART adherence [13]. Following the counseling sessions, a repeat VL test is performed. If the VL remains above 1000 copies/mL and adherence is confirmed, patients are moved to a second-line ART regimen [13].

For ART to be effective, lifelong adherence is required. Consistent ART adherence is crucial for successfully achieving viral suppression. Non-adherence often results in unsuppressed viral load, making individuals more vulnerable to other infections and increasing their mortality risk. Studies conducted in sub-Saharan Africa have identified numerous barriers that hinder ART adherence and make achieving VL suppression more difficult [14,15,16]. Commonly reported barriers to ART adherence include financial challenges, poverty, food insecurity, limited availability of healthcare resources, and transportation expenses. Despite substantial efforts to expand ART access and the implementation of a “treat all” approach, significant obstacles persist in the region, creating challenges in achieving VL suppression.

In this study, we aimed to identify socio-demographic, socioeconomic, clinical, virological, and immunological risk factors associated with treatment failure/success in sub-Saharan Africa, which was evaluated based on HIV RNA viral load suppression. Our study’s findings will contribute to a growing body of literature on the use of machine learning in healthcare, demonstrating how diverse algorithms can be applied to enhance predictive accuracy in health outcomes.

## 2. Materials and Methods

In this study, we undertook a secondary analysis of retrospective data collected through the Population-based HIV Impact Assessment (PHIA) surveys conducted in Zambia and Malawi. These assessments were part of the PHIA Project, funded by the U.S. President’s Emergency Plan for AIDS Relief (PEPFAR) and technically supported by the International Center for AIDS Care and Treatment Programs (ICAP) at Columbia University through the U.S. Centers for Disease Control and Prevention (CDC) [17,18,19]. The PHIA project surveys capture the status of the HIV epidemic in 13 sub-Saharan African countries and Haiti by assessing HIV prevalence, incidence, and viral load suppression at a population level [20]. The participants provided blood samples for HIV and other Sexually Transmitted Infections (STI) testing. Following the national testing standards for HIV in each country, the participants underwent in-home counseling and rapid HIV testing [20]. Written informed consent was obtained at every survey stage (head of the household interview, individual interview, and blood sample collection). All survey materials, including questionnaires and consent forms, were translated from English into local languages. The survey covered various aspects, including a household questionnaire, individual interviews addressing HIV care and treatment, HIV knowledge, behavioral risk factors associated with HIV incidence and prevalence, and questions on co-infections such as syphilis, tuberculosis, hepatitis B, and cervical cancer. Participants completed a standardized questionnaire, and whole blood samples were collected for HIV testing. HIV home-based testing and counseling (HBTC) was conducted following national guidelines, using a sequential rapid-test algorithm. This algorithm included the Determine HIV-1/2 test (Abbott Molecular Inc., Des Plaines, IL, USA) as the screening test and the Uni-Gold test (Trinity Biotech, Wicklow, Ireland) as the confirmatory test. Individuals with reactive results on both tests were identified as HIV-positive and referred to HIV care and treatment services. For participants identified as HIV-positive, further assessments were conducted, including CD4 T cell count measurement, HIV viral load testing, and the presence of antiretroviral drugs (ARVs). ARV presence was determined using high-resolution liquid chromatography-tandem mass spectrometry to detect markers such as efavirenz, atazanavir, lopinavir, and nevirapine. The methodology, including survey questionnaires, study design, and testing procedures, were previously described in detail in Sachathep K. et al. and the references therein [21].

The primary outcome of interest in this paper was HIV-1 RNA VL suppression, defined as having less than 1000 copies/mL [3]. The covariates examined included socio-demographic factors (such as age, gender, and urban residence), socio-economic factors (such as wealth index), ART status, duration on ART divided into four categories (not on ART, on ART for less than 12 months, on ART for 12–23 months, and on ART for 24 months or more), ART initiation, CD4 T cell count, and co-infections (such as hepatitis B and syphilis).

The objectives of the analyses were twofold: First, we sought to select a classifier model that will be used to evaluate the predictors of treatment failure/success in sub-Saharan Africa. The results from this analysis will inform the selection of the optimal predictive model based on a balance of accuracy, the area under the ROC curve (AUC), and interpretability. Second, we employed the selected ML classifier to identify predictors of HIV-1 RNA VL suppression, i.e., having less than 1000 copies/mL. The data we analyzed included clinical, socio-demographic, and socio-economic predictors such as CD4 count, age, gender, urban/rural residence, and wealth index to assess their impact on VL suppression along with ART duration, ART initiation 12 months or more prior to the survey, and ART treatment status. ART duration was categorized into the four following groups: (1) not on ART, (2) on ART for less than 12 months, (3) on ART for 12–23 months, and (4) on ART for more than 24 months. For our Zambia dataset, additional variables related to co-infections (syphilis and hepatitis B) were included. After data preparation and preprocessing, models were built and evaluated for classification accuracy and robustness, with the primary objective of comparing predictive performance across multiple machine learning algorithms. The dataset was refined by removing rows with missing values to maintain data quality and integrity in line with recommendations by Rubin (1987) on handling missing data in predictive modeling [22]. This procedure reduced the dataset to a size partitioned into training and testing subsets using a 0.75/0.25 split. This partitioning was accomplished using a random seed of 100 to ensure the replicability of results. The training dataset (75% of the data) was used for model fitting, while the testing dataset (25% of the data) enabled out-of-sample evaluation, an approach consistent with Hastie et al. (2009) [23]. Several ML algorithms were implemented to develop and compare predictive models for the targeted outcome variable, i.e., VL suppression. These algorithms included AdaBoost, AdaBag, Naive Bayes, Logistic Regression (Logistic), Neural Networks (MNet), Quadratic Discriminant Analysis (QDA), K-Nearest Neighbors (KNN), Random Forest (RF), Recursive Partitioning and Regression Trees (rpart), Support Vector Machines with Radial Basis Function Kernel (SVMRadial), and Linear Discriminant Analysis (LDA). Each model was implemented with cross-validation to ensure stable estimates of accuracy and minimize overfitting, as recommended by Kohavi (1995) [24]. The inclusion of multiple ML algorithms allowed for a comprehensive assessment of various classification approaches on the dataset. Each algorithm offers distinct advantages. For instance, ensemble methods such as Random Forest and AdaBoost are known for their high accuracy and robustness to overfitting, whereas simpler models like logistic offer greater interpretability, making them valuable in a clinical setting where model transparency is essential (Breiman, 2001) [25]. Each algorithm was trained on the training subset of the data using 10-fold cross-validation. The primary outcome metric was the Area Under Curve (AUC), calculated from the test data, which reflects the percentage of correctly classified cases. Performance evaluation was conducted on the test dataset to validate each model’s predictive power. Area under the ROC curve is a widely recognized metric for evaluating classification models, particularly when the outcome is binary, as in this analysis (Zou et al., 2007) [26]. Continuous and categorical patient characteristics were compared using the Wilcoxon rank sum and Fisher’s exact tests. Statistical analyses were performed using the R package version 4.4.2. All *p* values were two sided and considered statistically significant if <0.05.

## 3. Results

We analyzed data from 4820 ART-treated PLWH, of which 2239 are from Malawi and 2581 are from Zambia. The ages of the participants ranged from 15 to 59 years in Zambia and from 15 to 64 years in Malawi. In Malawi, 68.6% (n = 1536) of the participants achieved VL suppression. Meanwhile, 61% (n = 1562) achieved VL suppression in Zambia. These figures are less than the 95% suppression rate targeted by UNAIDS [6]. Most participants were females: 67.7% (n = 1516) in Malawi and 65.2% (n = 1684) in Zambia. The median age of participants was 40 years (IQR: 33–46 years) in Zambia, comparable to the median age of participants in Malawi, which was 39 years (IQR: 32–46 years). The median CD4 T cell count in Malawi was 485 cells/µL (IQR: 335.8–652.2), comparable to the median CD4 T cell count in Zambia, which was 453 cells/µL (IQR: 306.2–476.6). In Malawi, 55.6% (n = 1247) and in Zambia, 45.5% (n = 1176) of the participants initiated ART 12 months or more before the survey. Most of the participants from both countries were on ART. Only 33.8% (n = 758) and 39.3% (n = 1016) were not receiving ART in Malawi and Zambia, respectively. ART duration was divided into four categories in both countries: not on ART, on ART for less than 12 months, on ART for 12–23 months, and on ART for 24 months or more. A total of fifty-five percent (n = 1243) in Malawi and thirty-seven percent (n = 957) in Zambia were on ART treatment for 24 months or more. In Zambia, some participants were tested positive for co-infections like syphilis and hepatitis B, with 15.8% (n = 408) testing positive for syphilis and 6.6% (n = 171) testing positive for hepatitis B. In Malawi, the majority of the participants, 52.1% (n = 1167), resided in rural areas. On the other hand, in Zambia, the majority, 55.2% (n = 1427), are urban residents. The wealth index was categorized into three income levels (low, medium, and high), with a distribution ranging from 22.8% (n = 512) in the low-income group to 44.0% (n = 986) in the high-income group in Malawi. On the other hand, in Zambia, the distribution was more in the middle-income group, with 46.7% (n = 1206).

Table 1 presents the characteristics of our participants based on viral suppression status in Malawi. The participants with HIV suppression (median = 39 years, IQR = 32–46) were older than those without HIV suppression (median = 34 years, IQR = 28–41), showing a statistically significant difference (*p* < 0.001). Participants on ART medication had a higher viral suppression rate at 91.8% (n = 1410) than those without suppression at 12.5% (n = 88). Participants with HIV suppression have a higher CD4 T-cell count (median = 512 cells/µL, IQR = 366–675.5) compared to those without suppression (median = 319 cells/µL, IQR = 192–475), also showing a statistically significant difference (*p* < 0.001). The proportion of females who suppressed viral load is higher, with 71.1% (n = 1092), showing a statistically significant difference (*p* < 0.001). The proportion of patients who initiated ART before the survey was higher among those with suppression at 73.2% (n = 1124) than those without at 17.5% (n = 123), with a statistically significant difference (*p* < 0.001). The duration on ART varies, with 74% (n = 1137) of patients with suppression being on ART for more than 24 months, compared to 15.1% (n = 106) of patients without suppression (*p* < 0.001). Though statistically significant (*p* < 0.001), participants from urban residences achieved less viral suppression at 45% (n = 693) compared to those without suppression at 53.9% (n = 379). However, participants with a high income were more virally suppressed at 43% (n = 660), but it was not statistically significant (*p* = 0.57). In summary, our analyses indicate that age, female gender, CD4 T cell count, urban residence, initiation of ART 12 months or more before the survey, and ART duration were associated with HIV viral suppression in Malawi.

Table 2 presents the characteristics of patients based on viral suppression status in Zambia. The participants with VL suppression (median = 40 years, IQR = 33–46) were older than those without VL suppression (median = 33 years, IQR = 26–42), showing a statistically significant difference (*p* < 0.001). Participants on ART medication had a higher viral suppression rate at 87.2% (n = 1362) than those without suppression at 11.1% (n = 114), making ART status a statistically significant factor (*p*< 0.001). Participants with HIV VL suppression have a higher CD4 T-cell count (median = 473 cells/µL, IQR = 338–639) compared to those without suppression (median = 336 cells/µL, IQR = 210.5–490.2), a statistically significant difference (*p* < 0.001). Compared to Malawi, the proportion of females who suppressed VL was less in Zambia at 67.4% (n = 1053), and it was also not statistically significant (*p* = 0.02). The proportion of patients who initiated ART 12 months or more before the survey was higher among those with suppression at 67.2% (n = 1050) than those without at 12.4% (n = 126), but this difference was not statistically significant (*p* = 0.16). The duration on ART varies, with 55% (n = 904) of participants with suppression being on ART for more than 24 months compared to 51.3% (n = 523) of patients without suppression, making it statistically significant (*p* < 0.001). Conversely, the proportion of patients not on ART was lower among those with suppression at 12.7% (n = 199) than those without suppression at 80.2% (n = 817). Co-infections like syphilis and hepatitis B were more prevalent among those with suppression (16.1% for syphilis and 6.8% for hepatitis B) compared to those without (15.4% for syphilis and 6.3% for hepatitis B), showing no significant difference (*p* = 0.81). Participants from urban residences achieved more viral suppression at 57.9% (n = 904) with 51.3% (n = 523) of those from urban residences without suppression. However, participants who fell under the middle-income category were more virally suppressed at 46.1% (n = 720), compared to those without suppression at 47.7% (n = 486), showing a statistically significant difference (*p* < 0.001). In Zambia, our analyses identified that ART status, age, CD4 T-cell count, wealth index, and duration on ART were associated with VL suppression.

**Figure 1:**
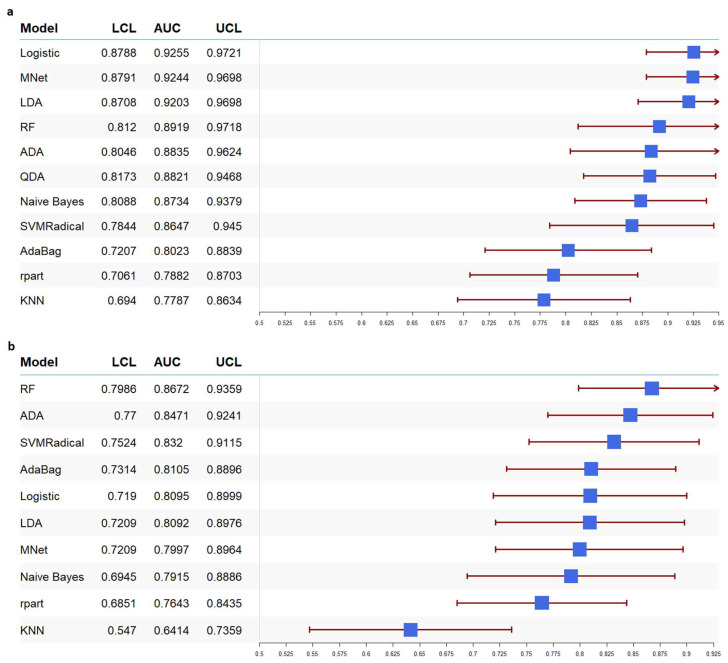
Forest plot showing the areas under the receiver operating characteristic curves with 95% confidence intervals for several machine learning classifiers in Malawi (**a**) and Zambia (**b**). Logistic: Logistic Regression, MNet: Neural Networks, LDA: Linear Discriminant Analysis, RF: Random Forest, ADA: AdaBoost, QDA: Quadratic Discriminant Analysis, Naive Bayes, SVMRadial: Support Vector Machines with Radial Basis Function Kernel, AdaBag, rpart: Recursive Partitioning and Regression Trees, and KNN: K-Nearest Neighbors. The blue box represents the point estimate of the models, while horizontal lines represent the confidence interval (CI). The ends of the lines mark the boundaries of the CI and arrow mark indicate the extended CI beyond the x-axis limits.

**Figure 2:**
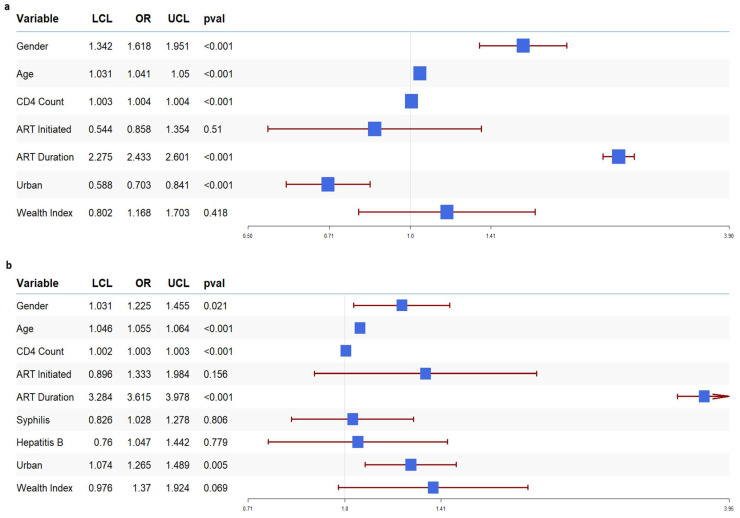
Forest plot of odds ratios (OR) together with their 95% confidence intervals for PLWH in Malawi (**a**) and Zambia (**b**). The blue box represents the point estimate of the analysis, while horizontal lines represent the confidence interval (CI). The ends of the lines mark the boundaries of the CI and arrow mark indicate the extended CI beyond the x-axis limits.

We applied various machine learning classifiers to our datasets to assess their predictive performance, with the ROC-AUC metric as the evaluation criterion. The results are shown in the forest plot in Figure 1. The AUC values ranged from 64% to 92% in both countries. In our Malawi data, as shown in Figure 1a, the Logistic classifier demonstrated the best performance with an AUC of 0.9255, followed by MNet with an AUC of 0.9244. This indicates that both models accurately identified VL suppression status in over 92% of the participants. In our Zambia data, as shown in Figure 1b, the Logistic classifier (AUC = 0.8095) was among the top classifiers to correctly identify VL suppression status in more than 80% of participants. Hence, we selected the Logistic classifier as the optimal predictive classifier model based on AUC values and interpretability. Thus, we ran univariate and multivariate logistic regression models to identify the socio-demographic, socioeconomic, clinical, and immunological factors associated with VL suppression in ART-treated PLWH. In both our Malawi and Zambia datasets, ART status was strongly associated with higher odds of viral suppression, for Malawi [OR = 98.07, 95% CI: 72.4–132.2, *p* < 0.01] and Zambia [OR =113.7, 95% CI: 84.9–152.2, *p* < 0.01]. The odds of viral suppression associated with the other variables are summarized in the forest plots presented in Figure 2. The forest plots of our data for both countries revealed that older age: Malawi [OR= 1.04, 95% CI: 1.03–1.05, *p* < 0.001] and Zambia [OR= 1.05, 95% CI: 1.04–1.06, *p* < 0.001]; higher CD4 T-cell count: Malawi [OR= 1.004, 95% CI: 1.003–1.004, *p* < 0.001] and Zambia [OR= 1.003, 95% CI: 1.002–1.003, *p* < 0.001]; and longer duration on ART: Malawi [OR= 2.43, 95% CI: 2.27–2.6, *p* < 0.001] and Zambia [OR= 3.61, 95% CI: 3.28–3.97, *p* < 0.001] were associated with higher odds of viral suppression. In Malawi, female gender [OR = 1.61, 95% CI: 1.34–1.95, *p* < 0.001] was also associated with viral suppression. Though it was not statistically significant, ART initiation 12 months or more before the survey in Malawi [OR = 0.86, 95% CI = 0.54–1.35, *p* = 0.51] and Zambia [OR = 1.33, 95% CI = 0.89–1.98, *p* = 0.15]; urban residence in Zambia [OR = 1.26, 95%CI: 1.07–1.48, *p* = 0.005]; female gender in Zambia [OR = 1.225, 95%CI:1.03–1.45, *p* = 0.021] and wealth index in Malawi [OR = 1.16, 95%CI: 0.80–1.70, *p* = 0.418] and Zambia [OR = 1.37, 95%CI: 0.97–1.92, *p* = 0.069] were also associated with viral suppression. Although co-infections are generally expected to complicate treatment and reduce the likelihood of viral suppression, our data showed no significant association between co-infections and VL suppression.

## 4. Discussion

This paper highlights the value of machine learning techniques in HIV/AIDS research, offering insights that can inform treatment strategies and improve patient outcomes. The study’s findings will contribute to a growing body of literature on the use of machine learning in healthcare, demonstrating how diverse and robust algorithms can be applied to enhance predictive accuracy in health outcomes. We undertook secondary analyses on our data collected from Zambia and Malawi. The objectives of the analyses were twofold: First, we selected the optimal predictive classifier model based on a balance of accuracy, the area under the ROC curve, and interpretability. Second, we employed the selected classifier model to identify predictors of HIV treatment success, assessed by HIV RNA viral suppression, in sub-Saharan Africa.

In our Malawi data, almost 69% of participants exhibited VL suppression, while in our Zambia data, 61% showed suppression. These rates are relatively lower compared to findings from previous studies conducted in other sub-Saharan African countries [27,28]. Besides treatment with ART, baseline CD4 T cell count, ART duration, and age were identified as important correlates of viral suppression. In our Malawi dataset, 91% of participants on ART medication achieved viral suppression, while in our Zambia dataset, 87% had similar outcomes. This significant impact of ART on viral suppression is consistent with previous research conducted across different regions of the world and populations [29,30,31], further validating our results and reinforcing the global consensus on the efficacy of ART treatment in suppressing VL. The median CD4 T cell count in our data from Malawi and Zambia was 485 cells/µL [IQR: 335.8–652.2] and 453 cells/µL [IQR: 306.2–476.6], respectively. These figures are slightly lower than the normal CD4 T-cell count range in PLWH. According to the WHO, a normal CD4 T-cell count range is from 500 to 1500 cells/mm^3^, progressively decreasing if ART treatment is not received [32,33]. In our data from both countries, higher CD4 T cell count was associated with higher odds of VL suppression, which is consistent with studies from sub-Saharan Africa as well as other regions globally [33,34,35,36]. We found that a longer duration on ART treatment was a significant predictor of VL suppression. In Malawi, individuals on ART for more than 24 months had a 74% suppression rate, compared to a 23% suppression rate for those on ART for less than 24 months. Similarly, for Zambia, 55% of participants on ART for 24 months or more achieved VL suppression compared to 37.5% on ART for less than 24 months. This association between ART adherence and VL suppression is consistent with the results reported in similar studies from sub-Saharan Africa [37]. Our findings show that a longer duration on ART and higher CD4 T-cell counts are also associated with VL suppression, which is consistent with other studies from sub-Saharan Africa and other regions globally [33,38,39,40,41]. Aligning with the results of other related studies from sub-Saharan Africa and different regions, our analysis demonstrates that older age was associated with higher odds of VL suppression [40,42,43].

Our findings from our Zambia dataset indicate that socio-economic factors, such as the wealth index, also play a role in achieving viral suppression, though are not statistically significant. This aligns with findings from previous studies conducted in sub-Saharan Africa [44]. This impact may be attributed to better access to treatment and educational programs among individuals in high-income groups compared to those in low- and middle-income groups [45]. Therefore, we would like to highlight the importance of awareness of HIV treatment among PLWH in the low-income index as an important factor for accessing ART to reach VL suppression targets set by UNAIDS. While urban/rural residence did not significantly affect VL suppression in our study, urban participants in our Zambia dataset showed better suppression than rural participants in our Malawi dataset. This disparity could be due to the higher number of rural participants in our Malawi dataset and the challenges rural areas face, such as limited access to healthcare, higher costs, and fewer educational resources on ART adherence [46].

The analyses in this paper provide valuable insights that can aid HIV clinicians and policymakers in enhancing strategies to combat HIV infection. Using the robust machine learning models, we highlight critical factors such as older age, CD4 T cell count, and ART duration, wealth index, urban residence as important factors affecting viral suppression. HIV/AIDS prevention programs in the regions should target and encourage PLWH to initiate ART early and adhere to ART treatment for effective treatment outcomes. We acknowledged the limitations of our study, and hence, our results must be interpreted cautiously. Compared with previous related studies, our study has many advantages. Unlike many previous studies, which were based either on a smaller sample size or specific groups such as female sex workers, MSM, or pregnant women, we utilized a large population-based survey, reducing the potential biases associated with small sample sizes and sampling specific groups of participants. It is important to interpret these findings cautiously due to certain limitations. One significant limitation is the retrospective data we used from a cross-sectional survey, which may not account for changes over time in key variables such as healthcare access or behavioral factors and variability in diagnostic standards or the standard CD4/CD8 ratio values across both regions. These disparities may influence the availability of ART regimens, patient adherence, and viral suppression outcomes, potentially limiting the generalizability of our findings. The self-reported nature of the data may still be affected by recall bias and the tendency to provide answers perceived as favorable, particularly on sensitive topics. Furthermore, while we categorized ART duration, the survey lacked details like the reasons for discontinuation or switching, which could have enriched our understanding of treatment effects. Lastly, the absence of long-term follow-up data restricts our ability to evaluate the durability of ART effects on viral suppression. Future research should incorporate prospective data collection and consider a more diverse set of variables to enhance the predictive accuracy of VL suppression models. Despite these limitations, our analysis underscores the importance of ART adherence, regular CD4 T-cell count monitoring, and comprehensive medication management, aligning with global goals such as the 95-95-95 targets. Ensuring patients are tested and treated with appropriate ART medications, achieving higher CD4 T-cell count, and adherence to ART treatment remains crucial for effective VL suppression.

## 5. Conclusions

In this paper, we utilized retrospective data from Zambia and Malawi and applied several machine-learning models to identify key factors influencing VL suppression in PLWH treated with ART. Our comprehensive analysis revealed that factors such as baseline CD4 T-cell count, duration on ART, and age significantly impact VL suppression. Though not statistically significant, ART initiation, wealth index, and urban/rural residence were also associated with VL suppression. The findings underscore the critical role of ART initiation, regular CD4 T-cell count monitoring, and strict adherence to medication regimens. Ultimately, ensuring comprehensive medication management and consistent monitoring were essential for achieving successful treatment outcomes and advancing toward global HIV suppression targets. Our analysis contributes to the growing body of evidence that supports integrated and holistic approaches to HIV care, which are vital for improving patient outcomes and achieving long-term VL suppression.

## Figures and Tables

**Table 1:** Variables by viral load suppression status among PLWH in Malawi.

Variable	Participants with HIV Suppressed (n = 1536, 68.6%)	Participants Without HIV Suppressed (n = 703, 31.4%)	*p*-Value
	N (%)	N (%)	
Age	38.5 (IQR: 46–32)	34 (IQR: 41–28)	<0.001
CD4 T-cell Count	512 (IQR: 675.5–366)	319 (IQR: 475–192)	<0.001
Gender (Female)	1092 (71.1)	424 (60.3)	<0.001
ART Initiation	1124 (73.2)	123 (17.5)	<0.001
ART Status	1410 (91.8)	88 (12.5)	0.54
ART Duration			
*Not on ART*	189 (12.3)	569 (80.1)	<0.001
*Less than 12 months*	0	0	
*12–23 months*	164 (10.7)	20 (2.8)	
*24 months or more*	1137 (74)	106 (15.1)	
Urban Residence	693 (45.1)	379 (53.9)	<0.001
Wealth Index			
*Low income*	356 (23.1)	156 (22.2)	0.57
*Middle income*	520 (33.9)	221 (31.4)	
*High income*	660 (43)	326 (46.4)	

**Table 2:** Variables by viral load suppression status among PLWH in Zambia.

Variable	Participants with HIV Suppressed (n = 1562, 61%)	Participants Without HIV Suppressed (n = 1019, 39%)	*p*-Value
	N (%)	N (%)	
Age	40 (IQR: 46–33)	33 (IQR: 42–26)	<0.001
Cd4 T-cell Count	473 (IQR: 639–338)	336 (IQR: 490.2–210.5)	<0.001
Syphilis	251 (16.1)	157 (15.4)	0.82
Hepatitis B	106 (6.8)	65 (6.3)	0.81
Gender Female	1053 (67.4)	631 (61.9)	0.02
ART Initiation	1050 (67.2)	126 (12.4)	0.16
ART Status	1362 (87.2)	114 (11.1)	<0.001
ART Duration			
*Not on ART*	199 (12.7)	817 (80.2)	<0.001
*Less than 12 months*	221 (14.1)	35 (3.4)	
*12–23 months*	167 (10.7)	10 (1)	
*24 months or more*	860 (55)	97 (9.5)	
Urban Residence	904 (57.9)	523 (51.3)	0.005
Wealth Index			
*Low income*	296 (18.9)	249 (24.5)	<0.001
*Middle income*	720 (46.1)	486 (47.7)	
*High income*	480 (30.7)	227 (22.3)	

## Data Availability

The dataset used in the manuscript is available from the Population-based HIV Impact Assessment (PHIA) Center: https://phia.icap.columbia.edu/ (accessed on 14 August 2024).
